# Trends in County-Level COVID-19 Incidence in Counties With and Without a Mask Mandate — Kansas, June 1–August 23, 2020

**DOI:** 10.15585/mmwr.mm6947e2

**Published:** 2020-11-27

**Authors:** Miriam E. Van Dyke, Tia M. Rogers, Eric Pevzner, Catherine L. Satterwhite, Hina B. Shah, Wyatt J. Beckman, Farah Ahmed, D. Charles Hunt, John Rule

**Affiliations:** ^1^Epidemic Intelligence Service, CDC; ^2^CDC COVID-19 Response Team; ^3^Office of the Assistant Secretary for Health, U.S. Department of Health and Human Services; ^4^Kansas Health Institute, Topeka, Kansas; ^5^Kansas Department of Health and Environment; ^6^Kansas Army National Guard.

Wearing masks is a CDC-recommended* approach to reduce the spread of SARS-CoV-2, the virus that causes coronavirus disease 2019 (COVID-19), by reducing the spread of respiratory droplets into the air when a person coughs, sneezes, or talks and by reducing the inhalation of these droplets by the wearer. On July 2, 2020, the governor of Kansas issued an executive order^†^ (state mandate), effective July 3, requiring masks or other face coverings in public spaces. CDC and the Kansas Department of Health and Environment analyzed trends in county-level COVID-19 incidence before (June 1–July 2) and after (July 3–August 23) the governor’s executive order among counties that ultimately had a mask mandate in place and those that did not. As of August 11, 24 of Kansas’s 105 counties did not opt out of the state mandate^§^ or adopted their own mask mandate shortly before or after the state mandate was issued; 81 counties opted out of the state mandate, as permitted by state law, and did not adopt their own mask mandate. After the governor’s executive order, COVID-19 incidence (calculated as the 7-day rolling average number of new daily cases per 100,000 population) decreased (mean decrease of 0.08 cases per 100,000 per day; net decrease of 6%) among counties with a mask mandate (mandated counties) but continued to increase (mean increase of 0.11 cases per 100,000 per day; net increase of 100%) among counties without a mask mandate (nonmandated counties). The decrease in cases among mandated counties and the continued increase in cases in nonmandated counties adds to the evidence supporting the importance of wearing masks and implementing policies requiring their use to mitigate the spread of SARS-CoV-2 (*1*–*6*). Community-level mitigation strategies emphasizing wearing masks, maintaining physical distance, staying at home when ill, and enhancing hygiene practices can help reduce transmission of SARS-CoV-2.

The Kansas mandate requiring the wearing of face coverings in public spaces became effective July 3, 2020. Data on county mask mandates were obtained from the Kansas Health Institute.^¶^ A Kansas state law** enacted on June 9, 2020, authorizes counties to issue public health orders that are less stringent than the provisions of statewide executive orders issued by the governor, which allowed counties to opt out of the state mask mandate. For this study, counties in Kansas that, as of August 11, 2020, did not opt out of the state mandate or adopted their own mask mandate were considered to have a mask mandate in place; those that opted out of the state mandate and did not adopt their own mask mandate were considered to not have a mask mandate in place.

Daily county-level COVID-19 incidence (cases per 100,000 population) was calculated using case and population counts accessed from USAFacts^††^for Kansas counties during June 1–August 23.^§§^ Rates were calculated as 7-day rolling averages. Segmented regression^¶¶^ was used to examine changes in COVID-19 incidence before and after July 3, 2020, among mandated and nonmandated counties. Mandated and nonmandated counties were compared to themselves over time, allowing for the control of constant county-related characteristics (e.g., urbanicity or rurality) that might otherwise confound a comparison between mandated and nonmandated counties. Sensitivity analyses were also conducted by 1) examining incidence trends after July 3 separately among mandated counties with and without other public health mitigation strategies and 2) recategorizing nonmandated counties that included cities mandating masks (n=6) as mandated counties. Analyses were conducted using SAS software (version 9.4; SAS Institute).

As of August 11, 24 (23%) Kansas counties had a mask mandate in place, and 81 did not. Mandated counties accounted for two thirds of the Kansas population (1,960,703 persons; 67.3%)*** and were spread throughout the state, although they tended to cluster together. Six (25%) mandated and 13 (16%) nonmandated counties were metropolitan areas.^†††^ Thirteen (54%) mandated counties and seven (9%) nonmandated counties had implemented at least one other public health mitigation strategy not related to the use of masks (e.g., limits on size of gatherings and occupancy for restaurants). During June 1–7, 2020, the 7-day rolling average of daily COVID-19 incidence among counties that ultimately had a mask mandate was three cases per 100,000, and among counties that did not, was four per 100,000 (Table). By the week of the governor’s executive order requiring masks (July 3–9), COVID-19 incidence had increased 467% to 17 per 100,000 in mandated counties and 50% to six per 100,000 among nonmandated counties. By August 17–23, 2020, the 7-day rolling average COVID-19 incidence had decreased by 6% to 16 cases per 100,000 among mandated counties and increased by 100% to 12 per 100,000 among nonmandated counties.

**TABLE:** Confirmed COVID-19 infection 7-day rolling average case counts, rates, and percentage changes, by mask mandate status*^,†^ and period — Kansas, June 1–August 23, 2020

Characteristic	Before executive order	Executive order effective^§^	After executive order	% Change in incidence^¶^	
	June 1–June 7	July 3–9	August 17–23	June 1–7 versus July 3–9	July 3–9 versus August 17–23
Mandated counties (N = 24)*^,^**					
No. of daily cases^††^	60	333	310	N/A	N/A
Incidence^§§^	3	17	16	467	–6
Nonmandated counties (N = 81)^†,^**					
No. of daily cases^††^	40	59	118	N/A	N/A
Incidence^§§^	4	6	12	50	100

**Abbreviations:** COVID-19 = coronavirus disease 2019; mandated = counties with a mask mandate; N/A = not applicable; nonmandated = counties without a mask mandate.

* Counties that as of August 11 did not opt out of the state mandate or adopted their own mask mandate shortly before or after the state mandate include Allen, Atchison, Bourbon, Crawford, Dickinson, Douglas, Franklin, Geary, Gove, Harvey, Jewell, Johnson, Mitchell, Montgomery, Morris, Pratt, Reno, Republic, Saline, Scott, Sedgwick, Shawnee, Stanton and Wyandotte. Total population in mask-mandated counties = 1,960,703 based on 2019 U.S. Census Bureau data.

^†^ Counties that took no official action to opt out of the state mask mandate or adopted their own mask mandate shortly before or after the state mandate were considered to have a mask mandate in place. Counties were considered to not have a mask mandate in place if they took official action to opt out of the state mask mandate and did not adopt their own mask mandate or if their official action used only the language of guidance (e.g., “should” or “recommend”). Total population in non–mask-mandated counties = 952,611 based on 2019 U.S. Census Bureau data.

^§^ Week of governor’s executive order (effective July 3, 2020).

^¶^ Change in incidence = [(incidence in period – incidence in previous period)/incidence in previous period] x 100.

** Data on county orders were collected through point-in-time surveys of local health department and other county officials and were supplemented with online searches for published orders and announcements on social media and local news sites. Text in the county orders was analyzed to determine whether mask mandates were in place as of August 11, 2020.

^††^ Seven-day rolling average number of new daily cases.

^§§^ Seven-day rolling average number of new daily cases per 100,000 population.

Trend analyses using segmented regression (Figure) indicated that during June 1–July 2, 2020, the COVID-19 7-day rolling average incidence increased each day in both counties that ultimately had mask mandates in place (mean increase = 0.25 cases per 100,000 per day; 95% confidence interval [CI] = 0.17–0.33) and counties that did not (mean increase = 0.08 cases per 100,000 per day; 95% CI = 0.01–0.14). After the governor’s executive order, COVID-19 incidence decreased each day in mandated counties (mean decrease = 0.08 cases per 100,000 per day; 95% CI = –0.14 to –0.03); in nonmandated counties, incidence continued to increase each day (mean increase = 0.11 cases per 100,000 per day; 95% CI = 0.01–0.21).

**FIGURE:**
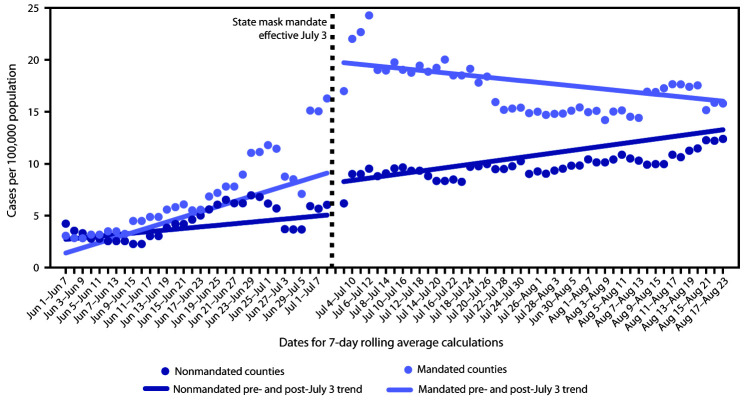
Trends* in 7-day rolling average of new daily COVID-19 cases per 100,000 population among mask-mandated^†^ and non–mask-mandated counties before (June 1–July 2)^§^ and after (July 3–August 23)^¶^ the governor’s executive order requiring masks — Kansas, June 1–August 23, 2020 **Abbreviation:** COVID-19 = coronavirus disease 2019. * Generalized estimating equation regression modeling with an autoregressive correlation variance structure was used to estimate trends over time within counties. Trends in 7-day rolling average of daily COVID-19 incidence among mask-mandated counties and non–mask-mandated counties were analyzed separately before (June 1–July 2, 2020) and after (July 3–August 23, 2020) the governor’s executive order requiring masks, effective July 3. ^†^ Kansas counties (n = 24) that as of August 11 did not opt out of the state mandate effective July 3, 2020, or adopted their own mask mandate shortly before or after the state mandate include Allen, Atchison, Bourbon, Crawford, Dickinson, Douglas, Franklin, Geary, Gove, Harvey, Jewell, Johnson, Mitchell, Montgomery, Morris, Pratt, Reno, Republic, Saline, Scott, Sedgwick, Shawnee, Stanton and Wyandotte. Data on county orders were collected through point-in-time surveys of local health department and other county officials and were supplemented with online searches for published orders and announcements on social media and local news sites. Text in the county orders was analyzed to determine whether mask mandates were in place as of August 11, 2020. Counties that took no official action to opt out of the state mask mandate or adopted their own mask mandate shortly before or after the state mandate were considered to have a mask mandate in place. Counties were considered to not have a mask mandate in place if they took official action to opt out of the state mask mandate and did not adopt their own mask mandate or if their official action used only the language of guidance (e.g., “should” or “recommend”). ^§^ Before the mask mandate (June 1–July 2), 7-day rolling average COVID-19 incidence increased each day (mean increase = 0.25 cases per 100,000 persons per day; 95% confidence interval [CI] = 0.17–0.33) in mask-mandated counties and increased each day (mean increase = 0.08 cases per 100,000 per day; 95% CI = 0.01–0.14) in nonmandated counties. ^¶^ After the mask mandate (July 3–August 23), 7-day rolling average COVID-19 incidence decreased each day (mean decrease = 0.08 cases per 100,000 persons per day; 95% CI = –0.14 to –0.03) in mask-mandated counties and increased each day (mean increase = 0.11 cases per 100,000 per day; 95% CI = 0.01–0.21) in nonmandated counties.

## Discussion

After implementation of mask mandates in 24 Kansas counties, the increasing trend in COVID-19 incidence reversed. Although rates were considerably higher in mandated counties than in nonmandated counties by the executive order, rates in mandated counties declined markedly after July 3, compared with those in nonmandated counties. Kansas counties that had mask mandates in place appear to have mitigated the transmission of COVID-19, whereas counties that did not have mask mandates continued to experience increases in cases.

The findings in this report are consistent with declines in COVID-19 cases observed in 15 states and the District of Columbia, which mandated masks, compared with states that did not have mask mandates (*7*). Mask requirements were also implemented as part of a multicomponent approach in Arizona, where COVID-19 incidence stabilized and then decreased after implementation of a combination of voluntary and enforceable community-level mitigation strategies, including mask requirements, limitations on public events, enhanced sanitation practices, and closures of certain services and businesses (*8*). The combining of community-level mitigation strategies including physical distancing and enhanced hygiene practices, in addition to consistent and correct use of masks, is a CDC-recommended approach.^§§§^ The decreased COVID-19 incidence among mask-mandated counties in Kansas occurred during a time when the only other state mandates issued were focused on mitigation strategies for schools as they reopened in mid-August. In at least 13 (54%) of the 24 mandated counties, the mask mandates occurred alongside other county-level recommended or mandated mitigation strategies (e.g., limits on size of gatherings and occupancy for restaurants), facilitating a potential synergistic effect resulting from combining community mitigation strategies. However, in sensitivity analyses, similar decreases in COVID-19 incidence after July 3 were observed among mandated counties with and without other mitigation strategies. Therefore, although implementing multiple mitigation strategies is the recommended approach, strategies related to mask use mandates appear to be important. Additional information on the utility and acceptability of mask mandates in public settings could help further inform health education campaigns aimed at increasing proper use of masks and strengthening mandate adherence.

The findings in this report are subject to at least four limitations. First, the ecologic design of this study and limited information on community mask-wearing behaviors and county implementation and enforcement provisions of mask mandates limit the ability to determine the extent to which the countywide mask mandates accounted for the observed declines in COVID-19 incidence in mandated counties. Second, this analysis did not account for mask ordinances in six cities in non–mask-mandated counties. However, in sensitivity analyses recategorizing nonmandated counties that included cities mandating masks as mandated counties, results were consistent with those in primary analyses, although they were attenuated. In those analyses, after the governor’s executive order, COVID-19 incidence among mandated counties stabilized rather than decreased, and incidence continued to increase among nonmandated counties. Third, although the design of this study limits potential confounding from constant county-related characteristics, the findings in this report are conditional on the absence of any time-varying factors (e.g., mobility patterns, changes in other community-level mitigation strategies, and access to testing) within counties before and after July 3. Nonetheless, in additional analyses examining testing data among Kansas counties during the study period, testing rates were observed to increase overall over time. Therefore, despite increases in testing during this period, decreases in COVID-19 incidence were observed in mandated counties after July 3. Finally, counties in Kansas with a mask mandate might not be representative of other U.S. counties. However, the findings are consistent with observations from other states that mask mandates are associated with declines in COVID-19 cases (*7*).

Masks are an important intervention for mitigating the transmission of SARS-CoV-2 (*1*–*6*), and countywide mask mandates appear to have contributed to the mitigation of COVID-19 spread in Kansas counties that had them in place. Community-level mitigation strategies emphasizing use of masks, physical distancing, staying at home when ill, and enhanced hygiene practices can help reduce the transmission of SARS-CoV-2.

**Summary**

**What is already known about this this topic?**

Wearing face masks in public spaces reduces the spread of SARS-CoV-2.

**What is added by this report?**

The governor of Kansas issued an executive order requiring wearing masks in public spaces, effective July 3, 2020, which was subject to county authority to opt out. After July 3, COVID-19 incidence decreased in 24 counties with mask mandates but continued to increase in 81 counties without mask mandates.

**What are the implications for public health practice?**

Countywide mask mandates appear to have contributed to the mitigation of COVID-19 transmission in mandated counties. Community-level mitigation strategies emphasizing use of masks, physical distancing, staying at home when ill, and enhanced hygiene practices can help reduce the transmission of SARS-CoV-2.

## Footnotes

* https://www.cdc.gov/coronavirus/2019-ncov/more/masking-science-sars-cov2.html?fbclid=IwAR28PppCa6x2uxwO8Z2baHM0KHS4JXx0inzzMQs3zRHV1qql_0a8mxZfpCw. https://www.cdc.gov/coronavirus/2019-ncov/prevent-getting-sick/cloth-face-cover-guidance.html.

† https://governor.kansas.gov/wp-content/uploads/2020/07/20200702093130003.pdf.

§ Allen, Atchison, Bourbon, Crawford, Dickinson, Douglas, Franklin, Geary, Gove, Harvey, Jewell, Johnson, Mitchell, Montgomery, Morris, Pratt, Reno, Republic, Saline, Scott, Sedgwick, Shawnee, Stanton, and Wyandotte counties. Data on county orders were collected through point-in-time surveys of local health department and other county officials and were supplemented with online searches for published orders and announcements on social media and local news sites. Text in the county orders was analyzed to determine whether mask mandates were in place as of August 11, 2020. Counties that took no official action to opt out of the state mask mandate or adopted their own mask mandate shortly before or after the state mandate were considered to have a mask mandate in place. Counties were considered to not have a mask mandate in place if they took official action to opt out of the state mask mandate and did not adopt their own mask mandate or if their official action used only the language of guidance (e.g., “should” or “recommend”).

¶ https://www.khi.org/policy/article/20-25. https://www.khi.org/assets/uploads/news/15015/august_11_update1105.pdf.

** https://ag.ks.gov/docs/default-source/documents/addendum-3-to-march-24-law-enforcement-duties-and-authorities-memo.pdf?sfvrsn = d088af1a_3.

†† https://usafacts.org/visualizations/coronavirus-covid-19-spread-map.

§§ August 23, 2020, was selected as the study end date because most Kansas counties had already started or were about to begin school the week of August 24, 2020. The implementation of in-person schooling would have signified an important change in events influencing COVID-19 incidence rates after the executive order.

¶¶ Generalized estimating equation regression modeling with an autoregressive correlation variance structure was used to estimate trends over time within counties. Trends in 7-day rolling average of daily COVID-19 incidence among mask mandated counties and among non–mask-mandated counties were analyzed separately before (June 1–July 2, 2020) and after (July 3–August 23, 2020) the governor’s executive order requiring masks, effective July 3.

*** Total population in mask-mandated counties = 1,960,703; total population in non–mask-mandated counties = 952,611; based on 2019 U.S. Census data.

††† As designated by the 2013 National Center for Health Statistics Urban-Rural Classification Scheme for Counties. https://www.cdc.gov/nchs/data_access/urban_rural.htm#Data_Files_and_Documentation.

§§§ https://www.cdc.gov/coronavirus/2019-ncov/community/community-mitigation.html.
