# *Erratum:* Vol. 69, No. 47

**DOI:** 10.15585/mmwr.mm695152a6

**Published:** 2021-01-01

In the report “Trends in County-Level COVID-19 Incidence in Counties With and Without a Mask Mandate — Kansas, June 1–August 23, 2020,” on p. 1777, the sixth footnote should have read “^††^ https://usafacts.org/visualizations/coronavirus-covid-19-spread-map. **Accessed August 31, 2020.**”

